# The *HAC1 *gene from *Pichia pastoris*: characterization and effect of its overexpression on the production of secreted, surface displayed and membrane proteins

**DOI:** 10.1186/1475-2859-9-49

**Published:** 2010-06-30

**Authors:** Mouna Guerfal, Stefan Ryckaert, Pieter P Jacobs, Paul Ameloot, Kathleen Van Craenenbroeck, Riet Derycke, Nico Callewaert

**Affiliations:** 1 Department of Biochemistry and Microbiology, Ghent University, Ghent, Belgium; 2 Department for Molecular Biomedical Research, VIB, Zwijnaarde, Belgium; 3 Department of Biomedical Molecular Biology, Ghent University, Ghent, Belgium; 4 Department of Dermatology, Brigham and Women's Hospital, Harvard Medical School, Boston, MA, USA; 5 Oxyrane Belgium, Zwijnaarde, Belgium; 6 Department of Physiology, Ghent University, Ghent, Belgium

## Abstract

**Background:**

The unfolded protein response (UPR) in eukaryotes upregulates factors that restore ER homeostasis upon protein folding stress and in yeast is activated by a non-conventional splicing of the *HAC1 *mRNA. The spliced *HAC1 *mRNA encodes an active transcription factor that binds to UPR-responsive elements in the promoter of UPR target genes. Overexpression of the *HAC1 *gene of *S. cerevisiae *can reportedly lead to increased production of heterologous proteins. To further such studies in the biotechnology favored yeast *Pichia pastoris*, we cloned and characterized the *P. pastoris HAC1 *gene and the splice event.

**Results:**

We identified the *HAC1 *homologue of *P. pastoris *and its splice sites. Surprisingly, we could not find evidence for the non-spliced *HAC1 *mRNA when *P. pastoris *was cultivated in a standard growth medium without any endoplasmic reticulum stress inducers, indicating that the UPR is constitutively active to some extent in this organism. After identification of the sequence encoding active Hac1p we evaluated the effect of its overexpression in *Pichia*. The *KAR2 *UPR-responsive gene was strongly upregulated. Electron microscopy revealed an expansion of the intracellular membranes in Hac1p-overexpressing strains. We then evaluated the effect of inducible and constitutive UPR induction on the production of secreted, surface displayed and membrane proteins. Wherever Hac1p overexpression affected heterologous protein expression levels, this effect was always stronger when Hac1p expression was inducible rather than constitutive. Depending on the heterologous protein, co-expression of Hac1p increased, decreased or had no effect on expression level. Moreover, α-mating factor prepro signal processing of a G-protein coupled receptor was more efficient with Hac1p overexpression; resulting in a significantly improved homogeneity.

**Conclusions:**

Overexpression of *P. pastoris *Hac1p can be used to increase the production of heterologous proteins but needs to be evaluated on a case by case basis. Inducible Hac1p expression is more effective than constitutive expression. Correct processing and thus homogeneity of proteins that are difficult to express, such as GPCRs, can be increased by co-expression with Hac1p.

## Background

**Figure 1:**
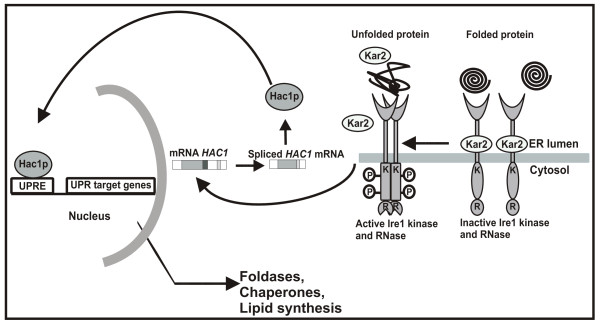
**The unfolded protein response in the yeast *S. cerevisiae***. Under normal conditions Ire1p is present in the ER membrane as a monomer in association with Kar2/Bip. Upon ER stress, in a first step, Kar2 dissociates from Ire1p which causes clustering of Ire1p in the ER membrane. In a second step, direct interaction of the unfolded protein with a stress sensing region of the Ire1p orients the cytosolic effector domains [4]. Clustering causes transautophosphorylation of the kinase domain (K) and simultaneous activation of the endoribonuclease (R) activity. Activation of Ire1p initiates an unconventional mRNA splicing reaction, which removes an intron from a unique mRNA species, *HAC1*, which encodes for an active transcription factor. Hac1p activates target genes coding for chaperones, foldases, lipid synthesis etc.

Secreted proteins enter the secretory pathway in the endoplasmic reticulum (ER), where they undergo co- and posttranslational modifications, such as glycosylation, phosphorylation, and the formation of disulfide bridges. Only correctly folded proteins leave the ER and proceed through the exocytic pathway. Accumulation of unfolded and misfolded proteins in the ER triggers the activation of the unfolded protein response (UPR). According to current models, the UPR in *S. cerevisiae *is a linear signaling pathway regulating the transcription of UPR target genes encoding chaperones, foldases, and proteins involved in glycosylation, lipid metabolism, *etc*.[1,2] (Fig. 1). The most prominent components regulating the UPR are the kinase/RNase, Ire1p and the transcription factor Hac1p. Under non-stress conditions Ire1p is a monomeric protein closely associated with the ER chaperone Bip/Kar2p [3]. As unfolded proteins accumulate in the ER lumen, Bip/Kar2p is released from Ire1p, which allows it to cluster and to interact with the unfolded proteins [4]. Clustering induces autophosphorylation of Ire1p and activates its RNase function. Activation of Ire1p initiates an unconventional splicing reaction of the *HAC1 *mRNA, at two specific sites different from the consensus intron sites recognized by the spliceosome.

*HAC1 *mRNA is constitutively expressed, but due to the secondary structure of the intron, no protein is produced when the mRNA remains unspliced [5]. Removal of the intron releases the translational block in the *HAC1 *mRNA. Finally, Hac1p is translocated to the nucleus, where it binds to UPR responsive elements (UPRE) in the promoter of UPR target genes. *HAC1 *homologues have been identified in mammals (*XBP1*), *C. elegans *(*XBP1*) and filamentous fungi (*HAC1/HACA*), and they undergo similar splicing reactions [6-8].

The methylotrophic yeast, *P. pastoris*, is widely used to express heterologous proteins. Different approaches are used to manipulate *P. pastoris *in order to increase the production of secreted proteins. As the UPR expands the ER's capacity of protein folding, attempts have been made to exploit this signal transduction pathway to augment recombinant protein expression. Several groups have described the effect of the constitutive expression of functional Hac1p from *S. cerevisiae *on the production of secreted heterologous proteins [9,10]. However, no study has reported the use of a *P. pastoris *Hac1p homolog because until now no *HAC1 *gene had been identified in this methylotrophic yeast. The aim of this study was to isolate the *HAC1 *gene from *Pichia pastoris *and to identify the intron that is spliced out to obtain functional Hac1p. After identifying the active Hac1p, we evaluated the effect of its overexpression in *Pichia *on the expression level of the UPR target gene Kar2p, on the morphology of intracellular membranes, and on growth of the yeast. We also evaluated the effect of Hac1p overexpression on the expression levels of surface displayed, secreted and membrane-bound heterologous proteins.

## Results and Discussion

### Identification of the *HAC1 *gene and its splice sites

**Figure 2:**
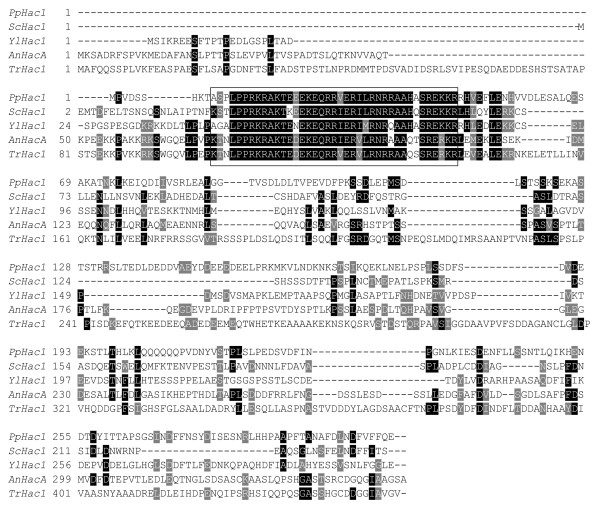
**Multiple sequence alignment of different Hac1p homologues**. Alignment of the amino acid sequence of Hac1p of *P. pastoris *(*Pp*), *S. cerevisiae *(*Sc*), *Y. lipolytica *(*Yl*), *A. nidulans *(*An*) and *T. reesei *(*Tr*). Only in the DNA binding bZIP domain similarity is observed (boxed).

The unconventional splicing of the *HAC1 *mRNA mediated by the transmembrane protein Ire1p plays a major role in activation of the UPR. Functional *HAC1 *homologues have been identified in *C. elegans*, mammals and filamentous fungi [6-8]. The sequence of the *HAC1 *gene from *P. pastoris *was found when searching for homologies to the *HAC1 *gene of *S. cerevisiae *in a draft of the *P. pastoris *genome (search kindly performed by James Cregg). A hit was found based on the homology between the relatively conserved DNA binding basic leucine zipper (bZIP) domains. Alignment of the AA sequence of *Pichia *Hac1p and other Hac1p homologues from yeast and filamentous fungi shows that there is no pronounced homology between the sequences, except in the bZIP domain (Fig. 2).

**Figure 3:**
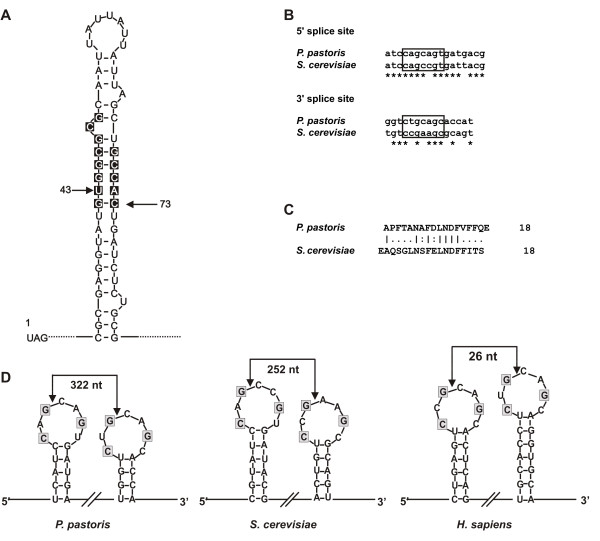
**Identification of the intron of the *HAC1 *mRNA**. A. Secondary mRNA structure of the 3'UTR of the *HAC1 mRNA*. The conserved nucleotides are highlighted in the figure. Conserved nucleotides are located in the distal end of the stem structure and are juxtaposed. B. Alignment of the 5' and 3' splice site of *P. pastoris *with the 5' and 3' border of the splice site of *S. cerevisiae*. Nucleotides in the box are present in the loop structure. C. New C-terminal domains are 18 amino acids in length and show conserved amino acids. D. Characteristic loop structures of the *HAC1 *mRNA of *P. pastoris*, *S. cerevisiae *and Human *XBP-1*. Predicted cleavage sites are indicated by an arrow, conserved nucleotides are boxed in grey.

Recently it was shown that a bipartite sequence in the 3' UTR of the *HAC1 *mRNA from *S. cerevisae *targets the unspliced mRNA to Ire1p for splicing [11,12]. The *HAC1 *3'UTR contains an extended stem-loop structure and two short sequences within this stem-loop are highly conserved in different *HAC1 *orthologues. A similar stem-loop structure was identified in the *Pichia HAC1 *3' untranslated region (Fig. 3A). As expected, conserved sequence motifs are juxtaposed in the distal part of the stem.

As the region around the splice sites of the *HAC1 *gene is relatively conserved, we were able to identify potential splice sites in the *P. pastoris HAC1 *gene by sequence similarity between the intronic regions of the *P. pastoris *and *S. cerevisiae HAC1 *genes (Fig. 3B). We predicted the splice sites to reside in characteristic stem-loop structures consisting of two seven-base rings resembling those found in *S. cerevisiae HAC1 *and *H. sapiens XBP1 *(Fig. 3D) [13,7]. We found that the intron consisted of 322 base pairs. The 5' splice site is situated in the coding region of the *HAC1 *gene, and splicing leads to replacement of the sequence encoding the C-terminal 45 AA by a sequence encoding 18 AA somewhat similar to the C-terminal part of the *S. cerevisiae *Hac1p (Fig. 3C).

The presence of an *IRE1 *homolog (XP_002493349) in the *Pichia *genome and the characteristics described above support an Ire1p mediated splicing reaction. Unspliced *HAC1 *mRNA is targeted to the Ire1p clusters via the bipartite region identified in the 3'UTR of the mRNA and splicing takes place at the identified stem-loop structures. The spliced *HAC1 *mRNA is translated to an active transcription factor which binds to UPRE (unfolded protein responsive elements) in UPR target genes. The best characterized UPRE, UPRE-1 (CANCNTG), is found in the promoter of the *Pichia HAC1 *gene (CAACTTG) and previously identified UPR target genes [14] such as *KAR2 *(CAGCGTG) and *INO1*(CAACTTG). The presence of an UPRE in the promoter of the *HAC1 *gene shows that Hac1p can up-regulate its own transcription as is also seen in *S. cerevisiae *[15].

### Confirmation of the splice sites

**Figure 4:**
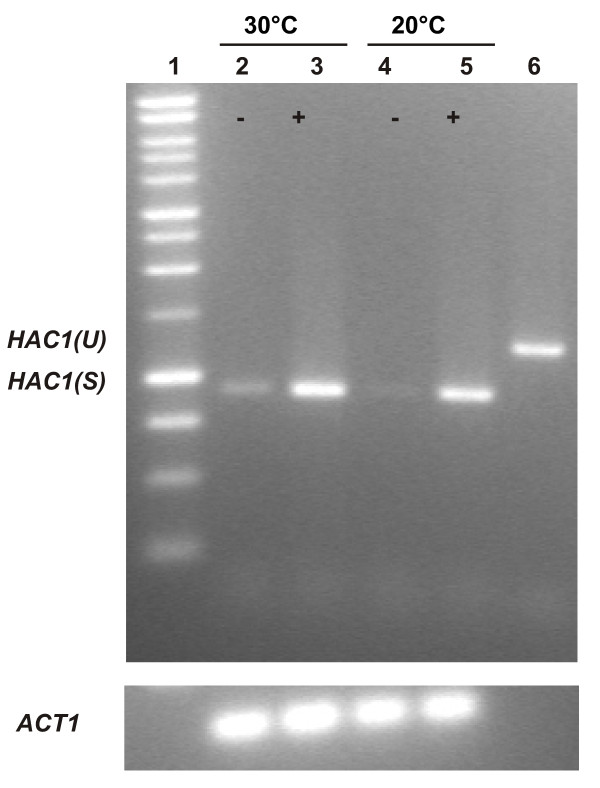
**RT-PCR on UPR induced and non-induced cultures**. The *HAC1 *mRNA is constitutively spliced. Also when the temperature is decreased from 30°C to 20°C we do not detect unspliced *HAC1 *mRNA (+ and - indicate growth in the presence or absence of 5 mM DTT respectively). Lane 6 is the result from a PCR performed on gDNA showing that the intron is clearly present in the genomic DNA sequence. The ladder on the gel is the 1 kb DNA marker from Promega.

To confirm the predicted splice sites, UPR was induced by adding dithiothreitol (DTT) to a *Pichia *culture in the mid-exponential growth phase. DTT disrupts the formation of disulfide bridges during folding in the ER, which results in accumulation of unfolded proteins. First strand cDNA was generated based on RNA fractions isolated from cultures in which UPR was either induced or not induced. An amplification product of the size expected for the spliced *HAC1 *mRNA was obtained for both the induced and the non-induced conditions (Fig. 4).

As this result shows that *HAC1 *mRNA splicing is constitutive in *P. pastoris*, it may indicate continuous folding stress on the growth temperature of 30°C used here. Therefore we also checked whether decreasing the growth temperature to 20°C alters the observed constitutive *HAC1 *mRNA splicing but no unspliced *HAC1 *mRNA was detected also at 20°C (Fig. 4). The amount of spliced *HAC1 *mRNA is however upregulated under stress conditions, indicating that ER stress regulates the transcription of *HAC1 *mRNA. The RT-PCR product was sequenced and its alignment with the *P. pastoris HAC1 *locus fully confirmed the predicted splice sites. We speculate that a basal splicing activity of the *HAC1 *mRNA and consequently a basal UPR activity might be one of the reasons why *Pichia *is a proficient protein secretor.

### Cloning and overexpression of the spliced *HAC1 *gene (*HAC(S)*)

To acquire the complete open reading frame of *P. pastoris HAC1(S)*, RT-PCR was performed on total RNA from a UPR induced culture. The cDNA fragment was cloned under control of the AOX1 promoter in the expression vector pBLHISIX. The resulting plasmid, pAOXHAC1, was transformed to *P. pastoris *GS115 cells and targeted into the *HIS4 *locus.

**Figure 5:**
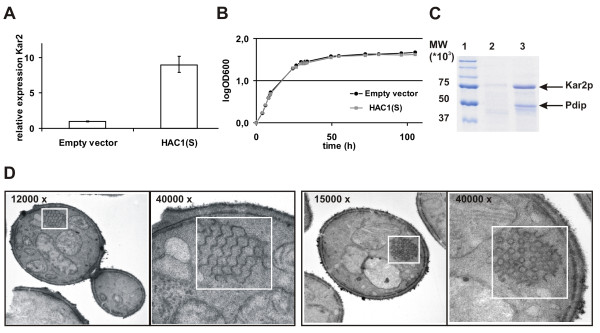
**Evaluation of artificial UPR induction**. A. Relative expression levels of the ER chaperone *KAR2 *determined by qPCR. The relative expression levels were calculated from the comparative threshold cycle values using the housekeeping actin gene as a control. B. Growth curve of a Hac1p overexpressing strain and an empty vector strain. Overexpression of Hac1p does not lead to a growth defect. C. Analysis of the culture supernatants after Hac1p induction. Lane 2 and 3 correspond to the empty vector and *HAC1(S) *transformed cells respectively. Two bands are prominently more abundant in the Hac1p overexpressing strain compared to the empty vector control. Mass spectrometry revealed that these bands correspond with Kar2p and Pdip, two ER HDEL-containing chaperones. D. EM pictures of a Hac1p overexpressing GS115 strain. Overexpression of Hac1p leads to the appearance of intracellular membranous structures (see white squares) with a cubic morphology.

After induction of *HAC1(S) *expression with methanol, transcriptional activation of the UPR was analyzed by quantifying *KAR2 *mRNA levels by qPCR. *KAR2 *is the best characterized UPR target gene. The expression level was nine-fold higher than in cells transformed with the empty vector, pBLHISIX (Fig. 5A). Besides observing upregulation of *KAR2 *mRNA, we also observed increased amounts of Kar2p and Pdip (identity confirmed by mass spectrometry, data not shown), which normally reside in the ER, in the culture supernatants of strains expressing *HAC1*(S) (Fig. 5C). This confirms reported findings: treatment with chemical agents that interfere with protein folding leads to secretion of Kar2p and other HDEL-containing proteins [16]. Kar2p is also secreted when cells overexpress heterologous proteins that induce UPR stress [17]. Secretion of HDEL-containing ER proteins could be a general characteristic of the UPR. It is possible that the maximum capacity of the ER retrieval mechanism is exceeded upon ER stress and proteins cannot be successfully retained anymore in the ER.

In contrast to previous reports, which showed that constitutive expression of active Hac1p slows the growth of *S. cerevisiae *[18,19], we did not observe a growth defect consequent to inducible expression of *P. pastoris HAC1(S) *(Fig. 5B).

The ER is the major site of lipid synthesis in the cell and can expand when considerable strain is placed on the secretory pathway. When unfolded and misfolded proteins accumulate in the ER, the ER needs to expand to accommodate them. This is reflected by increased biosynthesis of phosphatidyl-inositol and other lipids after UPR activation [20-23]. We investigated changes in intracellular membrane morphology by electron microscopy (EM) of *P. pastoris *cells overexpressing *HAC1(S)*. Cells were grown on methanol-containing medium for 48 h before being fixed for EM. In *P. pastoris*, we observed discrete regions of stacked membranes of seven or more well organized layers upon expression of *HAC1(S) *(Fig. 5D). The organization of the membranes differs from the membrane sheets observed in *S. cerevisiae *upon UPR induction. The membranes have a primitive cubic membrane morphology and it is the first time this membrane organization is observed in yeast. The fact that yeast can induce cubic membranes can be of great interest for the analysis of the biogenesis of such membranes.

### Expression of heterologous proteins

The production of large amounts of recombinant proteins is required for several pharmaceutical, biomedical and biotechnological applications, and so it is important to develop and optimize techniques to increase the yield of the proteins of interest. Overexpression of molecular chaperones is frequently employed to achieve this goal. As Hac1p overexpression leads to increased expression of the chaperones Bip/Kar2p, Pdip and others, co-expression of Hac1p has been explored as a means to increase expression of heterologous proteins. Constitutive expression of functional *S. cerevisiae *Hac1p leads to increased secretion of some homologous and heterologous proteins in S. *cerevisiae *[24,25], *P. pastoris *[9] and filamentous fungi [24]. We expressed homologous *Pichia *Hac1p in combination with heterologous proteins and evaluated the inducible expression of the transcription factor.

#### Surface displayed proteins

**Figure 6:**
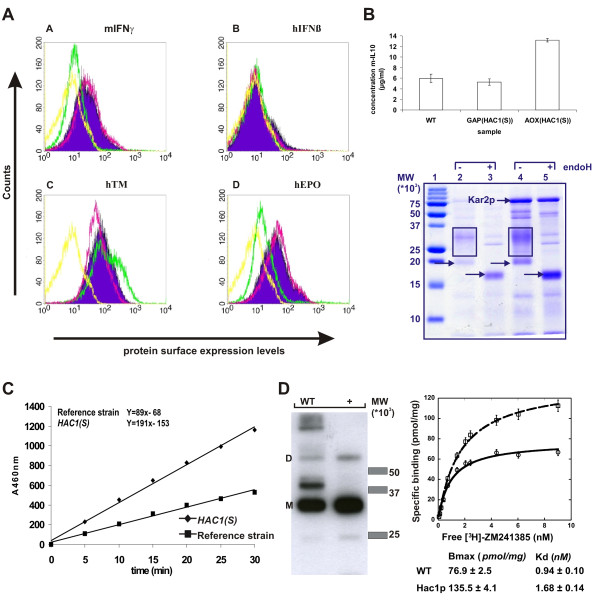
**Effect of overexpression *HAC1(S) *on the expression level of heterologous proteins**. A. Surface display: evaluation of Hac1p expression on the surface expression of heterologous proteins. Four proteins were evaluated: mouse interferon-γ (mIFNγ), human interferon-β (hIFNβ), human thrombomodulin (hTM) and human erythropoietin (hEPO). Yellow lines represent the non-displaying strains, purple the strain displaying the surface protein alone, yellow the strains transformed with inducible Hac1p and red the strains displaying the Hac1p constitutively. B. Secreted proteins, mIL-10: Comparison of mIL-10 production of a constitutive Hac1p expressing, inducible Hac1p expressing and the reference strain. The amount of IL-10 protein in culture supernatant was measured by ELISA. Coomassie gel of IL-10 production reference strain versus an inducible Hac1p expressing strain. Lane 2 and 3 illustrate the production of IL-10 protein (arrowheads) in the reference strain before and after endoH treatment respectively. Lane 4 and 5 show the production of the Hac1p overexpressing strain before and after endoH treatment. Kar2p is highly secreted (arrowheads) in the Hac1p expressing strain. Hyperglycosylation is highlighted in boxes. C. Secreted proteins, transialidase: Enzymatic hydrolysis of MUNANA to methyl-umbelliferon in function of time by trans-sialidase present in the medium. Enzymatic release of methyl-umbelliferon was determined every 5 min. D. Membrane proteins: Overexpression of Hac1p leads to a more homogenous dispersed Adenosine A2 receptor (M = monomer, D = dimer). Hac1p overexpression promotes a better processing of the α-mating factor. Radioligand binding studies on membranes of *P. pastoris *cells expressing the Adenosine A2 receptor with (broken line) and without (solid line) Hac1p.

To compare the effect of induced and constitutive overexpression of Hac1p on the expression of heterologous proteins, we used yeast surface display [26,27]. In this procedure, the protein of interest is fused to an endogenous yeast protein that is transported through the secretory pathway and which mediates covalent incorporation in the yeast cell wall. It has been demonstrated that the surface display level of a series of mutant single chain T cell receptors (scTCR) correlated well with soluble secretion levels, which suggests that the yeast surface display level could be used as readout for secretion efficiency [28]. Thus, we quantified the surface display levels of four heterologous proteins (mouse interferon-γ, human interferon-β, human thrombomodulin and human erythropoietin) in the presence or absence of HAC1p (expressed inducibly from the AOX1 promoter or constitutively from the GAP promoter) (Fig. 6A).

For the strains expressing the Hac1p protein constitutively, there was little or no improvement in surface expression levels compared to reference strains expressing the surface protein alone (Fig. 6A). For the strains overexpressing the inducible Hac1p, the expression level was either lower or higher, as follows: a) in the strain displaying mouse interferon-γ, the expression level was 1.8-fold lower; b) in the strain displaying human interferon-β, the low initial expression level was completely abolished; c) in the strain displaying human thrombomodulin, the expression level increased 1.9-fold compared to the reference strain, and d) in the strain displaying human erythropoietin, expression was 1.3-fold lower.

#### Secreted proteins

The spliced *HAC1 *cDNA under the control of the methanol-inducible AOX1 promoter was transformed to strains expressing mIL-10 or *Trypanosoma cruzi *trans-sialidase (TS) protein under control of the AOX1 promoter.

The expression of the mIL-10 protein in the absence or presence of constitutive or inducible expressed Hac1p was evaluated by ELISA. Results showed that comparable expression levels were obtained for the mIL-10 strain expressing the Hac1p constitutively (Fig. 6B). Co-expression of inducible Hac1p improved mIL-10 protein yield up to 2.2 times (Fig. 6B). Significant Kar2p secretion was observed in the strain expressing inducible *HAC1(S) *(Fig. 6B).

Secretion of trans-sialidase was assessed by measuring the enzymatic hydrolysis of 4-methyl-umbelliferyl-N-acetylneuraminic acid (MUNANA). TS strains expressing inducible HAC1p secreted up to 2.1-fold more TS than the reference strain (Fig. 6C). Coomassie stained gels showed that co-expression of Hac1p also here led to substantial secretion of Kar2p (data not shown).

#### Membrane proteins

To evaluate the effect of Hac1p overexpression on the expression of heterologous membrane proteins, we used the G-protein coupled receptor, Adenosine A2A receptor as a model. Expression of the receptor was analyzed by western blot, detecting the Rho1D4-tag present on the receptor. For the strains not overexpressing *HAC1(S)*, a prominent band appears of which the molecular weight corresponds to the monomeric receptor, accompanied with bands at higher molecular weight, probably corresponding to oligomers (Fig. 6D). Moreover a strong about 10 kDa higher band then the monomer is observed which is likely due to an incompletely processing of the pro-peptide in the α-mating factor leader sequence (9.3 kDa) which was used. Incomplete processing of the mating factor has been reported for other receptors expressed in *P. pastoris *[29]. However, very interestingly this higher molecular weight band is not present when the receptor is co-expressed with Hac1p, concomitant with an increased abundance of the band at the expected molecular weight of the monomer. It seems that co-expression of the A2A receptor with Hac1p leads to a more homogeneously dispersed receptor.

To test whether the adenosine A2A receptor is properly folded, radioligand binding studies on total membrane preparations were performed. These studies showed that the receptor fraction in the Hac1p-overexpressing strain can bind more ligand than the wild type strain (135.5 ± 4.1 pmol/mg *versus *76.9 ± 2.5 pmol/mg; Fig. 6D), a 1.8% increase is obtained, whereas its ligand affinity was reduced somewhat.

Seen the results of co-expression of Hac1p with heterologous proteins, we show that constitutive expression of functional Hac1p had only minor effects on the expression levels of proteins, if any at all. By contrast, inducible expression of Hac1p either increased or decreased the yield, depending on the protein evaluated.

Inducible expression of Hac1p might have reduced the expression of some proteins because different proteins may benefit from different levels of UPR induction, and in these instances the AOX1 promoter might have induced the UPR at an unsuitably high level. Moreover, UPR also activates the ER-associated degradation pathway (ERAD), which implies that if a protein's folding is not improved by the co-expression of chaperones, it will be targeted to the protein degradation pathway. This might also explain the observed decrease in the level of some proteins. The study of *HAC1(S) *under control of different promoters can be useful for optimizing chaperone co-expression.

We show that co-expression of Hac1p can be extremely useful when overexpressing membrane proteins for crystallography. A better processing of the α-mating factor is seen for the A2A receptor when coexpressed with Hac1p. This leads to a more homogenous receptor preparation which will facilitate crystal growth.

## Conclusion

From our studies we conclude that overexpression of Hac1p can be used as a technique to increase the production and the correct leader sequence processing of heterologous proteins but needs to be evaluated on a case by case basis. To express a single protein in heterologous hosts one has to overrule its specific bottleneck. Co-expression of chaperones to increase production levels is a trial and error process, but with substantial payoffs when successful. We also show that for GPCR's that are difficult to express, co-expression with Hac1p does not necessarily increase the amount of receptor but it can result in a more homogenously dispersed receptor which will facilitate crystallization for this class of proteins.

## Materials and methods

### Strains

MC1061 cells were used for the amplification of recombinant plasmid DNA. The *P. pastoris *strain GS115 (*his4*) was used to identify the *HAC1 *intron and to analyze the effect of overexpression of *HAC1(S) *on the cells. For the yeast surface display experiments, we used GlycoswitchMan5 strains [30] expressing fusion proteins under control of the inducible AOX1 promoter: human interferon-β with a-agglutinin; mature mouse interferon gamma with a-agglutinin; mature human erythropoietin with a-agglutinin; the lectin-like domain of mouse thrombomodulin with a-agglutinin. To evaluate the effect of the *HAC1(S) *co-expression on production of mouse IL-10 and trans-sialidase, we used a *P. pastoris *GS115 strain expressing mouse IL-10 and a *P. pastoris *GlycoSwitchMan5 strain expressing trans-sialidase, both under control of the AOX1 promoter. A *P. pastoris *GS115 strain expressing the A2A receptor provided with a Rho1D4-Tag under control of the AOX1 promoter was used to evaluate the effect of *HAC1(S) *on membrane protein expression.

### Media

Depending on the experimental settings, yeast strains were grown in YPD medium (10 g/L yeast extract, 20 g/L peptone, 20 g/L dextrose), BMGY (buffered Glycerol-complex Medium: 100 mM potassium phosphate pH 6.0 containing check 13.4 g/L YNB without amino acids, 10 g/l yeast extract, 20 g/L peptone and 10 g/L glycerol) or BMMY (Buffered Methanol-complex Medium: 100 mM potassium phosphate pH 6.0 containing 13.4 g/L YNB without amino acids, 10 g/L yeast extract, 20 g/L peptone and 10 g/L methanol). BMY was used as washing medium (100 mM potassium phosphate pH 6.0 containing 13.4 g/L YNB without amino acids, 10 g/L yeast extract and 20 g/L peptone).

### Plasmid construction

The intronless *HAC1 *cDNA was isolated from an UPR induced culture using forward primer 5'-GAATTCATGCCCGTAGATTCTTCTC-3' and reverse primer 5'-GCGGCCGCCTATTCCTGGAAGAATACAAAGTC-3', introducing an *Eco*RI site and a *Not*I site, respectively. The resulting PCR fragment was digested *Eco*RI and *Not*I and cloned behind the AOX1 or GAP promoter of the *Eco*RI/*Not*I opened plasmids pBLHISIX (J. Cregg) and pBLHISIX/GAP, respectively. The resulting plasmids were named pAOXHAC1 and pGAPHAC1.

### Strain construction

Competent *P. pastoris *cells were prepared and transformed by electroporation according to the protocol from the *Pichia *Expression kit (Invitrogen Cat. No. K1710-01). The pAOXHAC1 vector was linearized in the HIS4 gene to target the construct to the HIS4 locus for integration. Transformants were plated on RDB-HIS4 agar plates. Genomic DNA was prepared using the Epicenter Kit (Epicenter Biotechnologies, Madison, WI) and genomic integration was confirmed by PCR.

### UPR induction and identification of the splice site

Exponential phase cultures were incubated at 20°C or 30°C in the absence or the presence of 5 mM DTT for 1 h. RNA was isolated, treated with DNAse, and reverse transcribed using the iScript™cDNA Synthesis kit from Bio-Rad. Two microliters of cDNA was used in a PCR reaction with forward primer 5'-GAA TTC ATG CCC GTA GAT TCT TCT C-3' and reverse primer 5'-GCG GCC GCC TAT TCC TGG AAG AAT ACA AAG TC-3' (2 min 95°C, 30 sec 95°C, 30 sec 50°C, 1 min 30 sec 72°C; 40 cycles).

### Isolation of total RNA

Cells harvested in the exponential growth phase were washed once with sterile DEPC-treated water, and then 1 ml RNApure^® ^Reagent (Genhunter^® ^Corporation, Nashville, TN) and 1 g of baked glass beads were added. Cells were broken by vortexing 2 × 2 min using a Mixer Mill. The lysate was combined with 150 μl of chloroform, vortexed for 10 min, and centrifuged for 20 min at 13,000 rpm at 4°C. The upper phase was collected in a new tube and the RNA was precipitated with isopropanol on ice for 10 min. RNA was pelleted by centrifugation for 10 min at 13,000 rpm and 4°C and washed with 70% of ice-cold ethanol. The RNA was resuspended in 50 μl RNAse free water.

### Quantitative PCR

After DNase I digestion (RNase-free DNase Set Qiagen), 100 ng of RNA was reversed transcribed using the iScript™cDNA Synthesis kit from Bio-Rad. Template cDNA (corresponding to 25 ng RNA) was amplified in 25 μl containing 150 nM of the respective primers and 12.5 μl SYBR Green reaction buffer (Eurogentec). The absence of DNA contamination in RNA samples was tested by including RNA samples that had not been reverse transcribed. PCR conditions were as followed: 2 min at 50°C, 10 min at 95°C, followed by 40 cycles of 15 sec at 95°C, 20 sec at 60°C and 40 sec at 72°C. A melting curve was done to ensure that only a specific amplification product was obtained. Primer sequences were designed by Primer Express software (Applied Biosystems): Actin: 5'-GGTATTGCTGAGCGTATGCAAA-3' (forward) and 5'-CCACCGATCCATACGGAGTACT-3' (reverse); Bip/Kar2: 5'-CCAGCCAACTGTGTTGATTCAA-3' (forward) and 5'-GGAGCTGGTGGAATACCAGTCA-3' (reverse). The relative amounts of mRNA were calculated from the comparative threshold cycle values using the actin gene as a control.

### Growth curve

An overnight preculture in BMGY at 30°C was diluted in BMMY to an OD_600 _of 1 and incubation was continued. OD_600 _was measured at different times and logOD_600 _was plotted against time.

### Shake flask cultures (*HAC1(S) *induction and heterologous protein induction)

A pre-inoculum starting from a single colony was grown overnight in 5 ml of BMGY at 30°C and 220 rpm. OD_600 _was measured in the morning and the cells were diluted to an OD_600 _of 1 in a total of 12.5 ml BMGY in a 125 ml shake flask and grown for another 48 h. Cells were washed once with BMY, resuspended in BMMY, and induced for 48 h. Methanol (100%) was added every 8-12 h to a final concentration of 1% to maintain the induction. The adenosine A2A receptor was induced for 24 h before harvesting for analysis.

### mIL-10 ELISA

The amount of mouse IL-10 protein in the culture supernatants was measured by ELISA using Mouse IL-10 Cytoset (BIOSOURCE). Culture supernatants were diluted 1/4000, 1/8000, 1/12000 and 1/16000. Absorbance was measured at 450 nm with a reference absorbance of 650 nm (Thermo labsystems, Multiskan EX).

### Trans-sialidase assay

Trans-sialidase activity in the medium was measured by measuring the initial velocity of the hydrolysis of 4-methyl-umbelliferyl-N-acetylneuraminic acid (MUNANA) to fluorescent methyl-umbelliferon in a cytofluorometer (excitation and emission wavelengths at 360 nm and 460 nm, respectively). Twenty microliters of medium was added to 100 μl PBS containing 0.1% BSA and 500 μM MUNANA. Enzymatic release of methyl-umbelliferon was determined every 5 min. When fluorescence values are plotted as a function of time, the slopes of the linear curves allow comparison of the trans-sialidase activities in the media.

### Protein analysis

#### Mouse IL-10

OD_600 _was measured after induction. Proteins in supernatant corresponding to 2.8 × 10^7 ^cells were precipitated with DOC/TCA and separated in a 15% SDS-PAGE gel. Proteins were visualized by staining with Coomassie Brilliant Blue.

#### Adenosine A2A receptor

After induction, cells were centrifuged at 1500 ×g and the pellet was resuspended in ice-cold breaking buffer (50 mM sodium phosphate buffer pH 7.4, complete protease inhibitor (Roche), 5% glycerol). Cells were broken by vigorous vortexing with glass beads in a mixer mill for 10 × 1 min at 4°C. Cells were separated from the membrane suspension by low speed centrifugation (1000 ×g, 20 min, 4°C). Membranes were pelleted at 100,000 ×g and 4°C for 60 min and resuspended in resuspension buffer (50 mM sodium phosphate buffer pH 7.4 supplemented with a complete protease inhibitor from Roche) and snap-frozen in liquid nitrogen. The protein concentration of the membrane preparation was determined using the BCA reagent (Pierce, Rockford, IL) with BSA as a standard. Five micrograms of total membrane protein was analyzed by western blot. The blot was blocked overnight in blocking buffer (0.05% Tween-20 and 3% casein in 1× PBS) and probed with a 1/500 diluted primary mouse anti-Rho1D4 antibody, followed by a 1/3000 diluted secondary anti-mouse IgG peroxidase from sheep (Sigma Cat.n°NA931V). Protein bands were visualized with Renaissance western blot chemiluminescence reagent plus (PerkinElmer).

### Ligand binding studies A2A receptor

The procedures for studying binding at recombinant A2A receptors have been described [31]. Briefly, 5 μg of total membrane protein was incubated with different concentrations (0.06-14 nM) of the A2AR antagonist ^3^[H]ZM241385 in 500 μl binding buffer (20 mM HEPES pH 7.4, 100 mM NaCl). Adenosine deaminase (0.1 U) was added to degrade the adenosine released from the membranes and the membranes were incubated at 22°C for 1 h. Non-specific binding was determined in the presence of 10 mM theophylline. Measurements were performed in duplicate. After incubation, bound and free ligand were separated on Whatmann GF/B filters pretreated with 0.1% polyethylenimine using a Brandel cell harvester. The filters were washed three times with binding buffer and the amount of bound radioligand was measured on a liquid scintillation counter. The Kd and Bmax are determined by curve fitting using KaleidaGraph software (Synergy Software).

### Endo H treatment

mIL-10 protein was deglycosylated by treatment with 5 U endo H (New England Biolabs) according to the manufacturer's instructions. Supernatant samples (approximately 2.8 × 10^7 ^cells) from a methanol induced culture were combined with 5 U endo H and incubated overnight followed by precipitation with DOC/TCA and separation on a 15% SDS-PAGE gel.

### Electron microscopy

Samples were prepared for EM according to Baharaeen et al. [32]. Yeast cells were fixed for 2 h on ice in 1.5% paraformaldehyde and 3% glutaraldehyde in 0.05 M sodium cacodylate buffer, pH 7.2. After washing three times for 20 min in buffer, cells were treated with a 6% aqueous solution of potassium permanganate for 1 h at room temperature. After washing three times for 20 min in buffer, cells were dehydrated through a graded ethanol series, including bulk staining with 2% uranyl acetate at the 50% ethanol step, followed by embedding in Spurr's resin. Ultrathin sections of a gold interference color were cut using an ultra microtome (Ultracut E; Reichert-Jung), post-stained with uranyl acetate and lead citrate in a Leica ultrastainer, and then collected on formvar-coated copper slot grids. They were viewed with a transmission electron microscope 1010 (JEOL, Tokyo, Japan).

### Mass spectrometric analysis

For peptide mapping, 1 ml of culture supernatants (~ 9 × 10^8 ^cells) were precipitated with DOC/TCA, separated on SDS-PAGE, and stained with colloidal Coomassie Brilliant Blue.

The bands were cut out of the gel and washed with 100 μl of water, then with 100 μl of 50% acetonitrile in water, and then with 100 μl of 100% acetonitrile. The gel slices were dried in a SpeedVac centrifuge. Trypsin (50 ng) was added in a volume of 50 mM NH_4_HCO_3 _buffer (pH 8.0) that covered the slices, and the tubes were incubated overnight at 37°C. Then, 50 μl of acetonitrile was added, and the tubes were shaken well for 10 min. The supernatant was collected in a 500 μl tube, and the gel pieces were incubated a further 10 min in 30 μl of water; 50 μl of acetonitrile was added and the tube was shaken another 10 min. The second supernatant was combined with the first one, and the samples were vacuum evaporated to dryness. The dried material was reconstituted in 10 μl of 0.1% TFA in water and loaded on a C18 ZipTip (Millipore, Bedford, MA, USA) for desalting. Elution was done with 3 μl 50% AcCN containing 0.1% TFA. One microliter of the eluate was spotted on a location previously spotted with 1 μl of 4 mg/ml HCCA in 2:1 acetonitrile: water containing 0.05% TFA. Positive ion mode reflectron analysis was performed on an ABI4700 MALDI-TOF/TOF analyzer Bruker Reflex IV mass spectrometer tuned and calibrated in the 900-3500 m/z range. Peptide masses were searched with MASCOT against known *Pichia *protein sequences.

### FACS analysis

Precultures (5 ml) from positive clones were grown in YPD for 24 h. OD_600 _was measured and cultures were diluted to OD 1 in 2 ml BMGY in a 24-well plate and grown for 24 h, after which they were washed twice with distilled water and induced for 24 h in BMMY. Surface expression was demonstrated by indirect immunostaining with an antibody against the V5-epitope fused C-terminally to the V_H_H coding sequence. After induction, 10^7 ^cells in 1 ml PBS (pH 7.2) supplemented with 0.1% BSA (PBS/BSA), were incubated with 1 μl/ml anti-V5 antibody (1 μg/μl; Invitrogen), washed with PBS/BSA, and incubated with 1 μl/ml Alexa fluor 488-labeled goat anti-mouse IgG (1 μg/μl; Molecular Probes). After washing twice with PBS/BSA, the cells were analyzed on a BD FACS Calibur cytometer.

## Competing interests

The authors declare that they have no competing interests.

## Authors' contributions

MG identified the *HAC1 *intron, performed the studies on *HAC1(S) *overexpression, evaluated the effect of *HAC1(S) *overexpression on mIL-10 and on membrane proteins and drafted the manuscript. SR performed the surface display experiments. PJ assisted in the mIL10 experiments. PA completed the trans-sialidase assay. KVC helped in setting up the radioligand binding assays and reviewing the manuscript. RD prepared the EM pictures. NC reviewed the final manuscript. All authors read and approved the final manuscript.
